# GDF-15 Alleviates Hypoxia-Reoxygenation-Induced Damage to Human Placental Vascular Endothelial Cells by Regulating SIRT1

**DOI:** 10.7759/cureus.66073

**Published:** 2024-08-03

**Authors:** Cheng Chen, Jin Shi

**Affiliations:** 1 Department of Medical Sciences, Yangzhou Polytechnic College, Yangzhou, CHN; 2 Department of Gynecology and Obstetrics, Haimen People's Hospital, Haimen, CHN

**Keywords:** apoptosis, human placental vascular endothelial cells, sirt1, gdf-15, pregnancy-induced hypertension

## Abstract

Objective: Pregnancy-induced hypertension (PIH) is a common disease during pregnancy, which arises from maternal placental vascular endothelial cell dysfunction. Growth differentiation factor 15 (GDF-15) has a protective effect on the cardiovascular system. The purpose of this study is to explore the protective effect of GDF-15 against hypoxia-reoxygenation (H/R)-induced damage to human placental vascular endothelial cells (HPVECs) and the regulatory mechanism of SIRT1 in this effect.

Methods: Serum samples from healthy pregnant women and those with PIH were collected, and their GDF-15 and SIRT1 levels were examined. HPVECs were cultured in vitro and induced with H/R and GDF-15 at varying concentrations. The optimal concentration of GDF-15 in protecting HPVECs was determined by measuring cell viability via the CCK-8 assay. In H/R-induced HPVECs treated with GDF-15 and compound C (the AMPK inhibitor), expression levels of SIRT1, p-AMPK, and t-AMPK were detected. Cell apoptosis was examined by flow cytometry.

Results: Serum SIRT1 and GDF-15 were significantly higher in healthy pregnant women than in PIH patients. Suppressed viability and activated apoptosis in H/R-induced HPVECs were partially reversed by the treatment of GDF-15 at a concentration of 100 ng/mL. H/R induction significantly downregulated SIRT1 and p-AMPK in HPVECs, which were then upregulated by GDF-15. Moreover, the protective effect of GDF-15 on H/R-induced HPVECs was blocked by inhibiting the AMPK signaling pathway.

Conclusion: GDF-15 protects against H/R-inhibited cell viability and H/R-stimulated apoptosis in HPVECs by activating the AMPK signaling pathway to upregulate SIRT1.

## Introduction

Pregnancy-induced hypertension (PIH), a common disease during pregnancy, mainly arises from maternal endothelial cell dysfunction [1]. Sirtuin 1 (SIRT1) is a member of sirtuins to protect endothelial cells from senescence [2-4]. The role of SIRT1 in protecting from PIH-induced damage to the endothelium has been recently validated [5]. Growth differentiation factor 15 (GDF-15), one of the members of the transforming growth factor-β (TGF-β) superfamily, presents widely in cells and tissues. Its dysregulation is closely linked with inflammatory diseases, tumors, and cardiovascular diseases [6-9]. Downregulated GDF-15 in the circulatory system of patients with preeclampsia is indicative of a potential correlation between GDF-15 and PIH [10].

AMP-activated protein kinase (AMPK) is a signaling molecule that maintains endothelial homeostasis and protects cells from injury and stress [11]. Damages and apoptosis of placental vascular endothelial cells are involved in the pathological process of PIH [12]. Currently, hypoxia-reoxygenation (H/R) induction in human placental vascular endothelial cells (HPVECs) is an established approach to creating an in vitro PIH model [13]. In the present study, we aimed to explore the protective effect of GDF-15 against H/R-induced damage to HPVECs and the underlying mechanism.

## Materials and methods

Cell culture

HPVECs (ScienCell Research Laboratories, Wuhan, China) were cultured in the endothelial cell medium (ECM, Solarbio, Beijing, China) containing 10% of fetal bovine serum (FBS, Gibco, MA, USA) and placed in a cell incubator set at 5% CO_2_ and 37℃. Adherent cells were digested in trypsin-EDTA (0.25%, Thermo Fisher, MA, USA) and passaged for use. HPVECs at a concentration of 105/L were implanted and cultured until 60-70% adherence, followed by cell culture in serum-free ECM for 24 hours of synchronization.

H/R induction

Synchronous HPVECs at a 60-70% adherence were exposed to a constant hypoxic flow of 5% CO_2_ and 95% N2 for four hours, followed by reoxygenation in the normoxic environment containing 5% CO_2_ and 95% air for 6 hours.

CCK-8 assay

Synchronous HPVECs were implanted in a 96-well plate for adherence growth. Then, a fresh medium containing 10 μL of CCK-8 solution (Beyotime, Shanghai, China) was replaced. Optical density at 450 nm wavelength (OD450) was measured after four hours of cell culture using a microplate reader.

qRT-PCR

Total RNA was extracted from HPVECs using TRIzol and reversely transcribed to cDNA with the PrimeScript RT Reagent Kit at 37°C for 15 minutes and 85°C for five seconds. A qRT-PCR system composed of 1.0 μg total RNA, 2 μL of 5× PrimeScript RT Master Mix, and RNase-free distilled water to the recommended reaction volume of 20 μL was prepared. PCR was performed using the TaKaRa PCR Amplification Kit on the Roche real-time PCR system. Sequences of primers (Realgene, Nanjing, China) used in qRT-PCR were listed as follows: SIRT1, 5'-CAGACCTCCCAGACCCTCAAG-3' (forward) and 5'-TTCCTGCAACCTGCTCCAAG-3' (reverse); Glyceraldehyde 3-phosphate dehydrogenase (GAPDH), 5'-CTTCCAGGA GCGAGACC-3' (forward) and 5'-CGGAGATGATGACCCTTTT-3' (reverse).

Western blot

Total protein was extracted from HPVECs, and the prepared protein sample with 20 μg per lane was subjected to 10% SDS-PAGE and transferred on PVDF membranes. After inactivating non-specific antigens in TBST with 5% bovine serum albumin (BSA) for one hour at room temperature (RT), membranes were incubated with anti-GDF-15 (1:1000, PeproTech, NJ, USA), anti-SIRT1 (1:1000, Abcam), anti-phosphorylated AMPK (p-AMPK) (1:1000, Cell Signaling Technology, MA, USA), and anti-total-AMPK (t-AMPK) (1:1000 Cell Signaling Technology, MA, USA) overnight at 4℃. They were washed in TBST (15 minutes × three times) and reacted with HPR-labeled goat anti-rabbit or goat anti-mouse IgG antibody (ZSGB-BIO, Beijing, China) for one hour at RT. Enhanced chemiluminescence method (ECL) was used to expose Western blot bands and quantified by Image J. GAPDH (Boster, Wuhan, China) was used to normalize the protein level.

Flow cytometry

HPVECs were digested in trypsin (EDTA-free), washed in pre-cold PBS twice, and stained with Annexin V and PI (BD Biosciences, NJ, USA) in the dark. Within 15 minutes, the distribution of apoptotic cells was measured by flow cytometry.

Participants

A total of 30 healthy pregnant women at a gestational age of 24-36 weeks receiving a regular prenatal examination at Haimen People's Hospital, Haimen, Jiangsu, China, between March 2023 and March 2024, were randomly enrolled in our study. During the same period, 30 PIH patients at a gestational age of 24-36 weeks were allocated. Pregnant women with essential hypertension, chronic kidney disease, diabetes mellitus, infectious diseases, and immune rheumatism or using antihypertensive drugs were excluded. Fasting serum samples were collected from all participants for measuring serum SIRT1 and GDF-15 using the commercial Enzyme-Linked Immunosorbent Assay (ELISA) kits (Abcam, Cambridge, UK). Approved by the Ethics Committee of Haimen People's Hospital (approval no. 2023-KY01), ethical principles of the medical research complied with the Declaration of Helsinki.

Statistical processing

Measurement data in a normal distribution were expressed as mean ± standard deviation (SD). Differences between groups were evaluated by the independent samples t-test, while those among three or more groups were compared by one-way analysis of variance (ANOVA) and the Q-test. A significant difference was determined by a p-value of less than 0.05.

## Results

Serum SIRT1 and GDF-15 decrease in PIH patients

**Figure 1:**
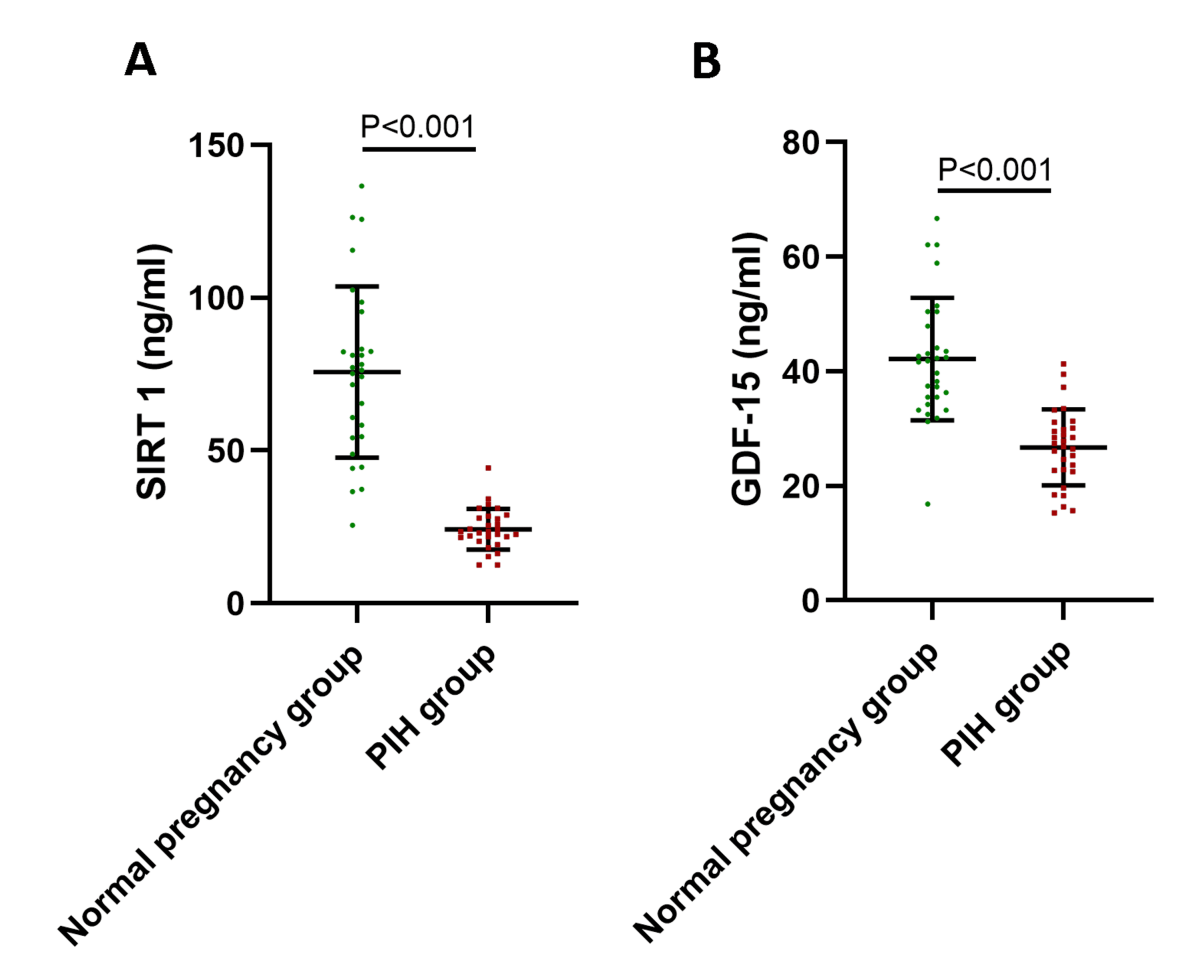
Serum SIRT1 and GDF-15 of healthy pregnant women and PIH patients. (A) Serum SIRT1 of healthy pregnant women and PIH patients. (B) Serum GDF-15 of healthy pregnant women and PIH patients. SIRT1, sirtuin 1; GDF-15, growth differentiation factor 15; PIH, pregnancy-induced hypertension

Serum SIRT1 (75.54 ± 27.5 ng/mL vs. 24.04 ± 6.67 ng/mL) and GDF-15 (42.13 ± 10.69 ng/mL vs. 26.73 ± 6.61 ng/mL) were significantly lower than those of healthy pregnant women (both p < 0.05, Figure 1).

GDF-15 increases the viability of H/R-suppressed HPVECs

**Figure 2:**
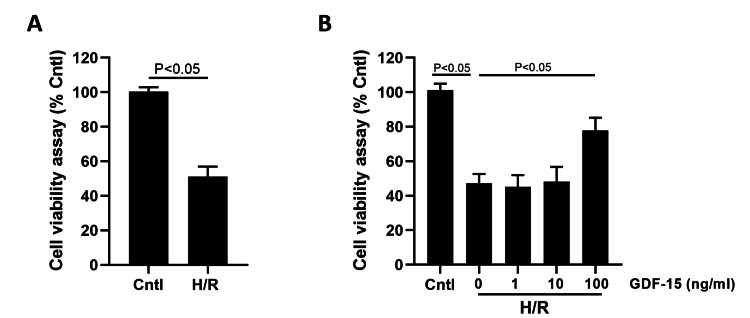
GDF-15 increased the viability of H/R-suppressed HPVECs (A) Cell viability in H/R-induced HPVECs and those with blank control. (B) Cell viability in blank control, H/R-induced HPVECs, and H/R-induced HPVECs treated with 1 ng/mL, 10 ng/mL, and 100 ng/mL GDF-15. GDF-15, growth differentiation factor 15; HPVECs, human placental vascular endothelial cells; H/R, hypoxia-reoxygenation

H/R induction significantly reduced the viability of HPVECs (Figure 2A). In H/R-induced HPVECs intervened by 1 ng/mL, 10 ng/mL, and 100 ng/mL GDF-15, the suppressed viability was most significantly reversed by 100 ng/mL GDF-15 (Figure 2B). As a result, an intervention of 100 ng/mL GDF-15 was adopted in the following in vitro experiments.

GDF-15 protects against H/R-induced damage to HPVECs by regulating SIRT1 and AMPK

**Figure 3:**
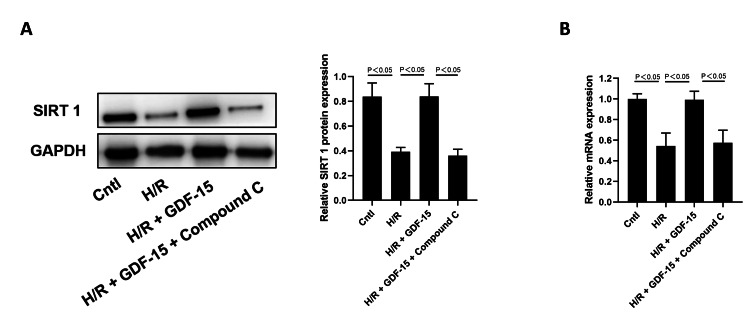
GDF-15 reversed H/R-induced downregulation of SIRT1 in HPVECs. (A) SIRT1 protein in blank controls, H/R-induced HPVECs, and H/R-induced HPVECs treated with GDF-15. (B) SIRT1 mRNA in blank controls, H/R-induced HPVECs, and H/R-induced HPVECs treated with GDF-15. GDF-15, growth differentiation factor 15; HPVECs, human placental vascular endothelial cells; SIRT1, Sirtuin 1; H/R, hypoxia-reoxygenation

H/R induction in HPVECs significantly downregulated the mRNA and protein levels of SIRT1, which were then partially reversed by GDF-15. Interestingly, the regulatory effect of GDF-15 in reversing H/R-induced downregulation of SIRT1 in HPVECs was blocked by the AMPK inhibitor compound C (Figure 3).

GDF-15 inhibits H/R-induced apoptosis of HPVECs

**Figure 4:**
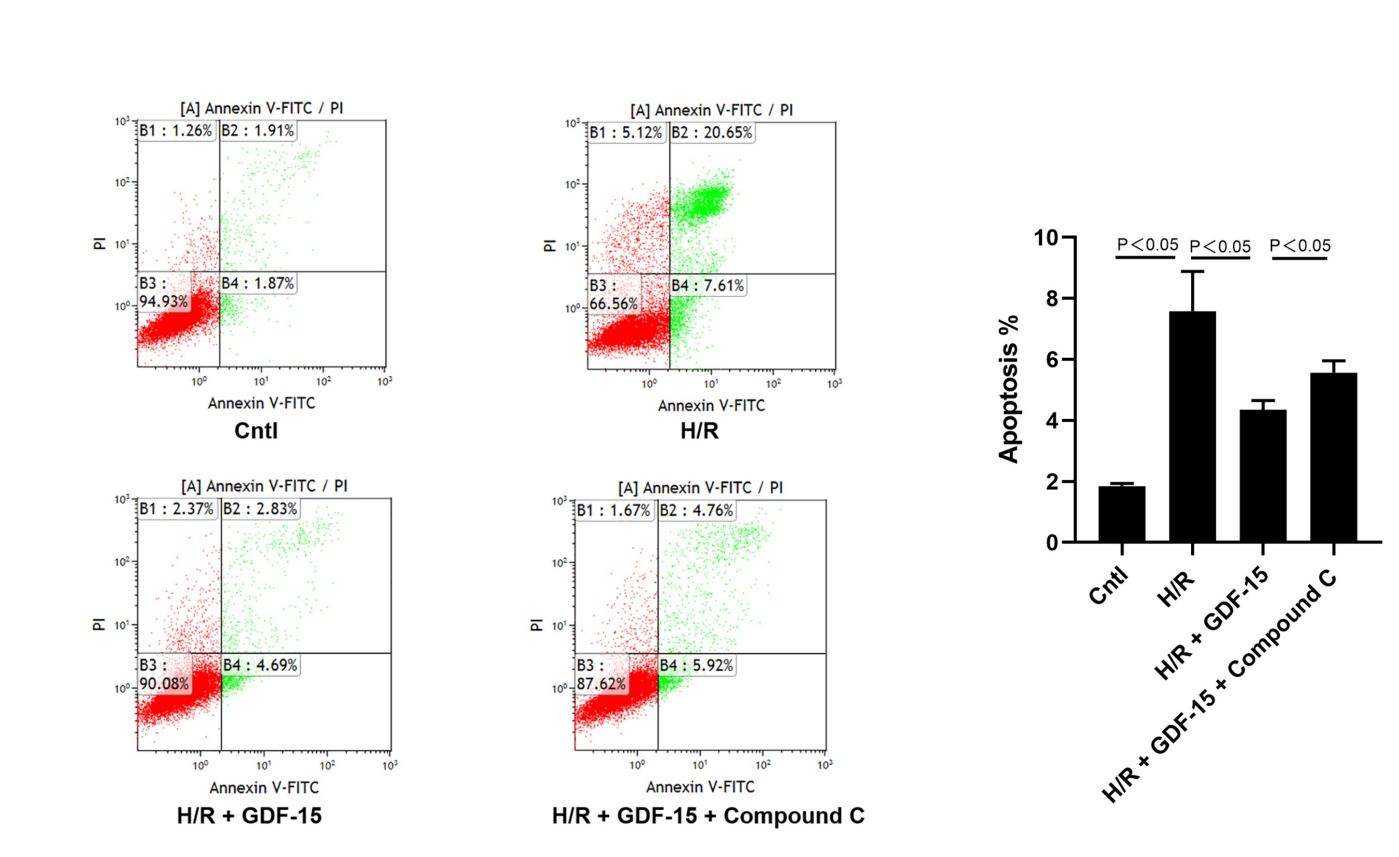
GDF-15 inhibited H/R-induced apoptosis of HPVECs. Apoptotic rate in blank control (1.84% ± 0.08%), H/R-induced HPVECs (7.53% ± 1.31%), H/R-induced HPVECs treated with GDF-15 (4.35% ± 0.29%), and H/R-induced HPVECs treated with GDF-15 plus compound C (5.56% ± 0.38%). GDF-15, growth differentiation factor 15; HPVECs, human placental vascular endothelial cells; H/R, hypoxia-reoxygenation

Compared with that of blank control, H/R induction significantly increased the apoptotic rate in HPVECs (7.53% ± 1.31% vs. 1.84% ± 0.08%, p < 0.05). GDF-15 significantly inhibited the H/R-induced apoptosis of HPVECs, although its protective role was impaired by the intervention of AMPK inhibitor Compound C (p < 0.05, Figure 4).

GDF-15 protects against H/R-induced damage to HPVECs via the AMPK signaling pathway

**Figure 5:**
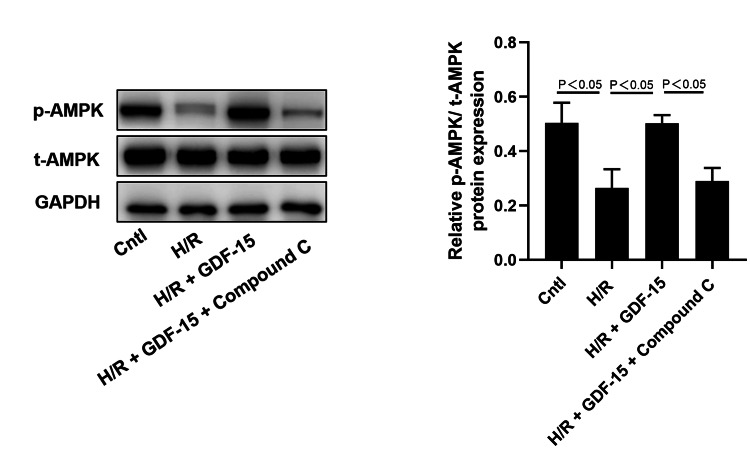
GDF-15 protects against H/R-induced damage to HPVECs via the AMPK signaling pathway. Protein expression levels of p-AMPK and t-AMPK in the blank control, H/R-induced HPVECs, H/R-induced HPVECs treated with GDF-15, and H/R-induced HPVECs treated with GDF-15 plus compound C. GDF-15, growth differentiation factor 15; HPVECs, human placental vascular endothelial cells; H/R, hypoxia-reoxygenation

In H/R-induced HPVECs, the p-AMPK having been significantly downregulated was found partially reversed after the treatment of GDF-15. Nevertheless, the role of GDF-15 in upregulating p-AMPK during H/R-induced damage was buffeted by compound C (Figure 5).

## Discussion

PIH is a condition occurring after 20 weeks of gestation in women with previously normal blood pressure. It affects 6-10% of pregnant women, leading to a high risk of maternal or neonatal death [14]. The pathogenesis of PIH is linked with placental abnormalities, and clinical symptoms of PIH gradually resolve after placental delivery. Placental vascular growth ensures the normal transfer of oxygen and nutrients from the maternal blood across the placenta to the fetus. During the processes of placental blood circulation and nutrition transport, HPVECs are of profound significance in controlling blood pressure within a normal range [15,16]. Potential damages to HPVECs eventually pull the trigger to PIH [13]. In the present study, H/R induction significantly inhibited the viability and stimulated the apoptosis of HPVECs in vitro.

SIRT1 is a NAD+-dependent deacetylase equipped with inhibitory effects on oxidative stress, aging, and inflammatory responses [5]. Loss of SIRT1 suppresses the migration and proliferation of trophoblasts [5]. Mice with SIRT1 knockout present symptoms similar to PIH, suggesting the potential involvement of SIRT1 in PIH [17]. In a clinical cohort, plasma SIRT1 is found lower in PIH patients than in healthy pregnant women [18]. We consistently revealed a significantly lower serum SIRT1 in PIH patients and lower mRNA and protein levels of SIRT1 in H/R-induced HPVECs.

GDF-15, also known as macrophage inhibitory cytokine-1 (MIC-1), is a member of the TGF-β superfamily orchestrating regenerative inflammation [19-21]. Benes et al. [22] unveiled a correlation of serum GDF-15 with the prognosis of chronic kidney disease combined with heart failure. Overexpression of GDF-15 fights against oligomycin-induced neuronal cell damage [20]. An increased level of circulating GDF-15 serves as the key mechanism underlying the role of metformin in maintaining energy metabolism balance and controlling body weight [23]. During the first trimester, a declined GDF-15 level is associated with the risks of miscarriage and ectopic pregnancy [24,25]. Chen et al. [9] suggested that the overexpression of GDF-15 may alleviate PIH. Our findings showed a significantly higher serum GDF-15 in healthy pregnant women than in PIH patients. In vitro findings illustrated the role of GDF-15 in reversing the suppressed viability and activated apoptosis in H/R-induced HPVECs. More importantly, downregulated SIRT1 in H/R-induced HPVECs was partially reversed by the treatment of GDF-15.

**Figure 6:**
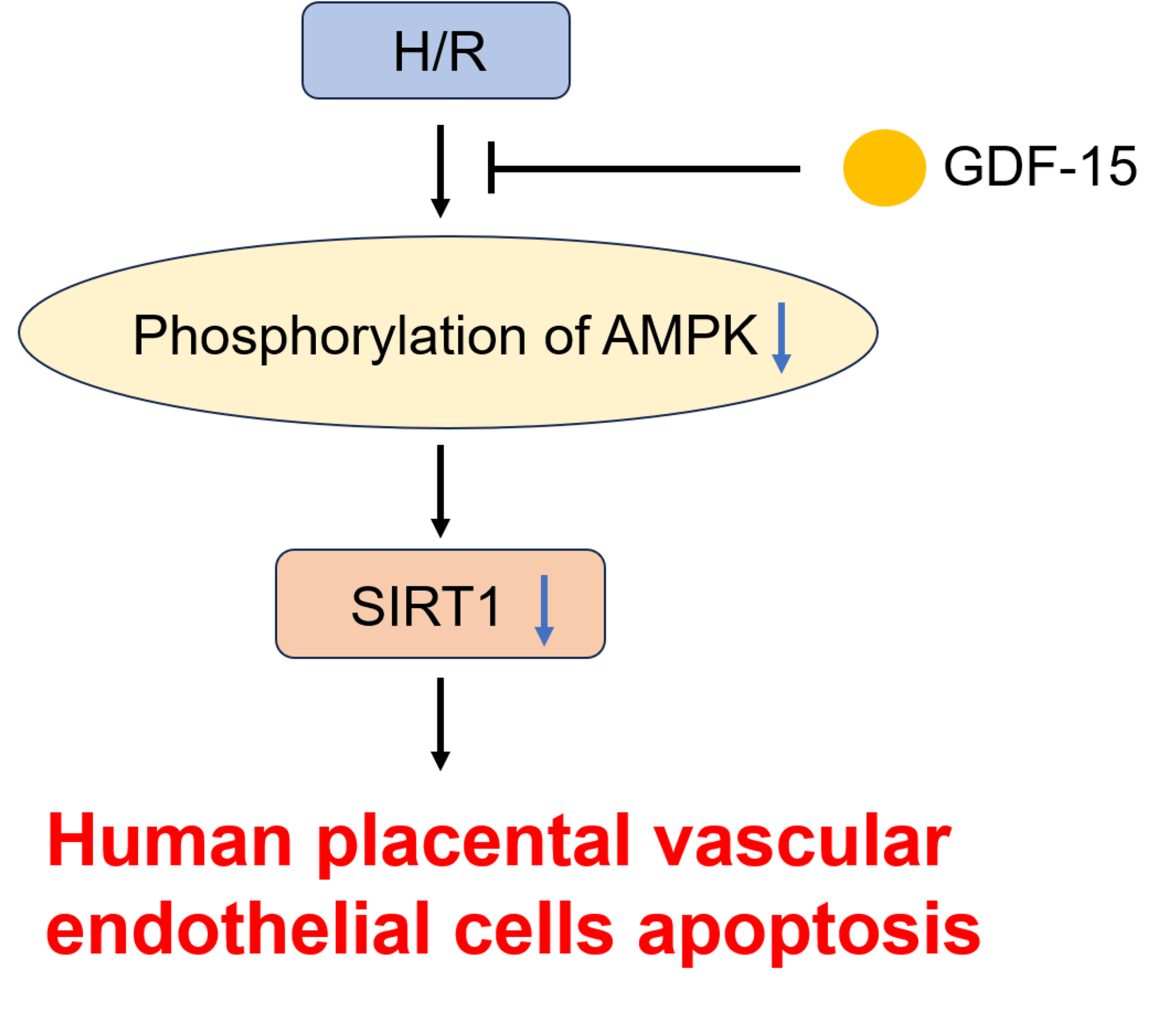
Schematic view of the damage effect of H/R on HPVECs. H/R downregulates SIRT1 expression by inhibiting AMPK phosphorylation, ultimately causing HPVECs apoptosis. GDF-15 protects HPVECs from H/R injury by promoting AMPK phosphorylation and inhibiting downregulation of SIRT1 expression. GDF-15, growth differentiation factor 15; HPVECs, human placental vascular endothelial cells; H/R, hypoxia-reoxygenation

AMPK acts as a cellular energy sensor and regulator of metabolic homeostasis. The AMPK signaling transduction participates in maintaining endothelial homeostasis and protecting endothelial cells against damage and stresses [26]. Zheng et al. [27] reported the involvement of the AMPK signaling pathway in H_2_O_2_-induced damage to human umbilical vein endothelial cells. The activated AMPK underpins the protective function of metformin in cardiac microvascular endothelial damage [28]. CTRP9 alleviates H/R-induced damages to HPVECs by activating the AMPK signaling pathway, suggesting the involvement of AMPK in the pathogenesis of PIH [29]. Our data showed that GDF-15 upregulated SIRT1 in H/R-induced HPVECs by overexpressing p-AMPK (Figure 6). The protective effect of GDF-15 on H/R-induced HPVECs reversed by AMPK inhibitor compound C further validated the significant function of AMPK in PIH.

Limitations

The present study had certain limitations. First, conclusions were not tested in animal experiments. If it can also be demonstrated in animal experiments that GDF-15 has a protective effect on PIH, the evidence to support our conclusions would be stronger. Second, to detect the serum concentrations of SIRT1 and GDF-15 in PIH patients, we collected serum from 30 PIH patients. However, it was a single-center study with a small sample size, which may cause potential biases. Our findings require further validation in multi-center, prospective controlled studies and animal experiments.

## Conclusions

Taken together, we reported the lower serum levels of SIRT1 and GDF-15 in PIH patients. GDF-15 greatly protected against H/R-induced inhibition of viability and activation of apoptosis in HPVECs by upregulating SIRT1 and activating AMPK. GDF-15 may serve as a therapeutic target for PIH. Our findings should be further validated in in vivo experiments.
